# Edukation bei chronischen Kopfschmerzen durch Übergebrauch von Schmerz- und Migränemitteln: Ergebnisse einer systematischen Literaturrecherche

**DOI:** 10.1007/s00482-025-00891-9

**Published:** 2025-06-18

**Authors:** Hans-Christoph Diener, M. Graf, H. Löhr, P. Kropp

**Affiliations:** 1 Abteilung für Neuroepidemiologie, Institut für Medizinische Informatik, Biometrie und Epidemiologie (IMIBE), Universität Duisburg-Essen, Hufelandstraße 55, 45147 Essen, Deutschland; 2 Lundbeck GmbH, Hamburg, Deutschland; 3 Institut für Medizinische Psychologie und Medizinische Soziologie, Universitätsmedizin Rostock, Rostock, Deutschland

**Keywords:** Migräne, Kopfschmerz, Medikamentenübergebrauch, Medikamentenübergebrauchskopfschmerz, Edukation, Migraine, Headache, Medication overuse headache, Medication overuse, Education

## Abstract

**Hintergrund:**

Kopfschmerzen durch Übergebrauch von Schmerz- und Migränemitteln („medication overuse headache“ [MOH]) treten häufig bei Patienten mit chronischer Migräne auf und sind gekennzeichnet durch eine Verschlechterung der Migräne bei gleichzeitig zunehmender Verwendung von Akutmedikation. Chronische Kopfschmerzen durch Übergebrauch von Schmerz- und Migränemitteln sind ein relevantes Problem in der klinischen Praxis und die aktuelle Leitlinie empfiehlt, dass Patienten durch eine Edukation angeleitet werden sollen, ihren Medikamentenübergebrauch zu reduzieren.

**Ziel:**

Ziel war es, zu untersuchen, welche publizierten Ansätze zur Edukation bei chronischen Kopfschmerzen durch Übergebrauch von Schmerz- und Migränemitteln vorliegen.

**Methodik:**

Es wurde eine systematische Literaturrecherche in PubMed durchgeführt. Eingeschlossen wurden Publikationen, in denen inhaltliche Details zu Edukationen bei chronischen Kopfschmerzen durch Übergebrauch von Schmerz- und Migränemitteln aufgeführt sind. Die beschriebenen Edukationsansätze wurden thematisch analysiert.

**Ergebnisse:**

Es wurden 12 Publikationen eingeschlossen. Die beschriebenen Edukationsmaßnahmen waren wirksam zur Reduktion oder Prävention von Medikamentenübergebrauch oder chronischen Kopfschmerzen durch Übergebrauch von Schmerz- und Migränemitteln. Die Edukationsinhalte sind heterogen, wobei bestimmte Kernthemen in den meisten Edukationsansätzen enthalten sind. Diese lassen sich den Themenfeldern „Grundlagen zum Konzept Medikamentenübergebrauch oder chronischen Kopfschmerzen durch Übergebrauch von Schmerz- und Migränemitteln“, „Risikofaktoren“ und „Therapieoptionen“ zuordnen. In wenigen Publikationen fanden sich zusätzlich inhaltliche Schwerpunkte, die sich dem Themenfeld „Absprache von Behandlungszielen“ zuordnen lassen.

**Schlussfolgerungen:**

Edukation ist wirksam bei der Prävention und Behandlung von Patienten mit Medikamentenübergebrauch oder chronischen Kopfschmerzen durch Übergebrauch von Schmerz- und Migränemitteln, wird aber uneinheitlich umgesetzt. Die hier präsentierte Übersicht kann als Grundlage für einen strukturierten Edukationsansatz für die Behandlung von chronischen Kopfschmerzen durch Übergebrauch von Schmerz- und Migränemitteln dienen.

## Abstract (en)

**Background:**

Medication overuse headache (MOH) is often found in patients with chronic migraine as a result of worsening migraine accompanied by increasing amounts of acute medications. MOH is a relevant problem in clinical practice and the current German guideline on MOH recommends educational intervention to guide patients to reduce acute medication use.

**Objectives:**

The aim was to investigate published approaches for education in MO (medication overuse) and MOH.

**Methods:**

A systematic literature search was conducted in PubMed. Publications were included in which details for education for the prevention and treatment in MO or MOH were described. The educational approaches were analyzed thematically.

**Results:**

Twelve publications were included in this analysis. The described educational measures were effective in reducing or preventing MO or MOH. The educational content was heterogeneous, but some common topics were included in most of the educational approaches. These can be categorized in “education on the OH or MOH concept”, “risk factors” and “treatment options”. In few publications, additional topics related to “agreement on treatment” were mentioned.

**Conclusion:**

Education is effective in patients with medication overuse or medication overuse headache, but approaches differ. The overview presented here can be used as a basis to develop a structured educational approach for MOH.

## Einleitung

Chronische Kopfschmerzen durch Übergebrauch von Schmerz- und Migränemitteln („medication overuse headache“ [MOH]) sind ein verbreitetes Problem bei Kopfschmerzpatienten mit einer globalen Prävalenz zwischen 0,7 und 1 % [4]. Betroffen sind insbesondere Patienten mit hochfrequenter episodischer (HFEM) oder chronischer Migräne (CM), bei bis zu 70 % der Patienten mit chronischer Migräne sind auch Kopfschmerzen durch Übergebrauch von Schmerz- und Migränemitteln vorhanden [3].

Chronische Kopfschmerzen durch Übergebrauch von Schmerz- und Migränemitteln sind nach den Kriterien der International Headache Society (IHS) definiert als Kopfschmerz an 15 oder mehr Tagen pro Monat bei einem Patienten mit einer vorbestehenden primären Kopfschmerzerkrankung, der sich als Folge eines regelmäßigen Übergebrauchs von Kopfschmerzakutmedikation für mehr als 3 Monate entwickelt. Als Übergebrauch wird, je nach Medikament, die Einnahme an mindestens 10 (Triptane, Mutterkornalkaloide, analgetische Mischpräparate) oder 15 Tagen (Nichtopioidanalgetika) pro Monat bezeichnet. Dieser Kopfschmerz verschwindet meist, aber nicht immer nach Beendigung des Übergebrauchs [4, 6].

Chronische Kopfschmerzen durch Übergebrauch von Schmerz- und Migränemitteln sind mit starken Einschränkungen und einer Reduktion der Lebensqualität verbunden [1]. Zusätzlich ist Medikamentenübergebrauch ein Risikofaktor für eine Krankheitsprogression der Migräne [16, 28]. Darum ist es dringend notwendig, Patienten mit Medikamentenübergebrauch oder chronischen Kopfschmerzen durch Übergebrauch von Schmerz- und Migränemitteln dabei zu unterstützen, den Übergebrauch zu beenden.

Die aktuelle S1-Leitlinie zu chronischen Kopfschmerzen durch Übergebrauch von Schmerz- und Migränemitteln empfiehlt eine Patientenedukation mit dem Ziel, die Einnahme von Akutmedikation zu reduzieren [4]. Edukation allein kann bei einem Teil der Patienten bereits ausreichend sein, um einen Medikamentenübergebrauch oder chronische Kopfschmerzen durch Übergebrauch von Schmerz- und Migränemitteln erfolgreich zu behandeln oder zu verhindern [4]. In der vorliegenden Arbeit wurde anhand einer systematischen Literaturrecherche untersucht, welche konkreten Edukationsansätze bei chronischen Kopfschmerzen durch Übergebrauch von Schmerz- und Migränemitteln bisher publiziert wurden. Die Edukationsmaßnahmen wurden auf ihre Inhalte hin untersucht und anschließend diskutiert.

## Methoden

Es wurde eine systematische Suche in PubMed mit den folgenden Begriffen durchgeführt: migraine, medication overuse, triptan overuse, medication overuse headache, education, advice, awareness, brief intervention, training. Aus den Suchergebnissen wurden alle Publikationen eingeschlossen, die eine Edukation für Patienten mit Medikamentenübergebrauch oder chronischen Kopfschmerzen durch Übergebrauch von Schmerz- und Migränemitteln inhaltlich beschrieben. Ausgeschlossen wurden Artikel, in denen eine Edukation erwähnt, aber nicht näher dargestellt wurde, Sekundärpublikationen einer bereits anderswo beschriebenen Edukationsintervention sowie Artikel in anderen Sprachen als Deutsch oder Englisch. Eingeschlossene Studien wurden auf die Themenfelder der beschriebenen Edukation hin untersucht.

## Ergebnisse

**Abb. 1:**
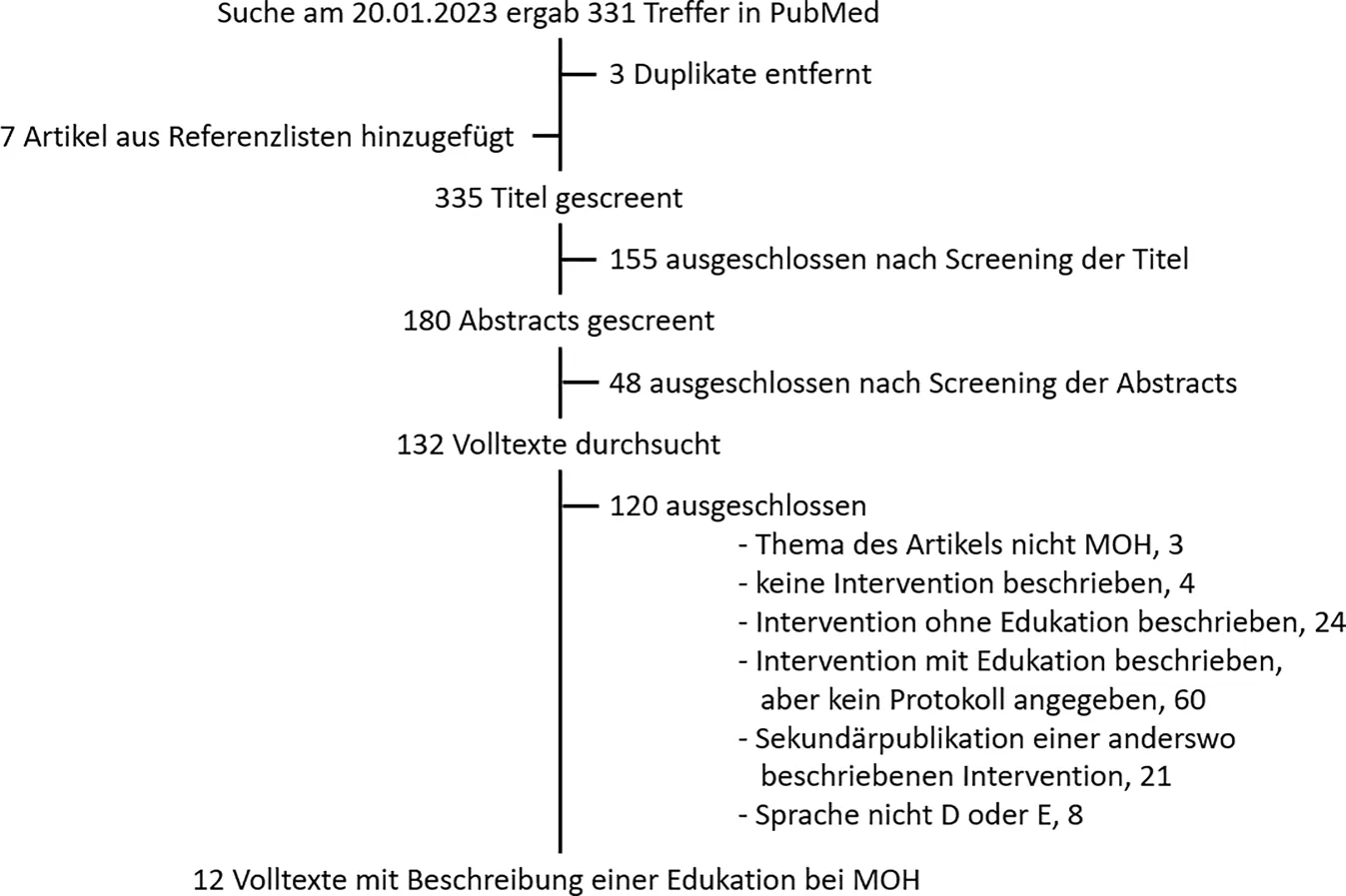
Literaturfluss. *MOH* chronische Kopfschmerzen durch Übergebrauch von Schmerz- und Migränemitteln

Aus 331 Suchergebnissen wurden 12 Artikel mit konkreten Angaben zu einer Edukation von Patienten mit Medikamentenübergebrauch oder chronischen Kopfschmerzen durch Übergebrauch von Schmerz- und Migränemitteln identifiziert (Abb. 1). Eine Übersicht über die 12 relevanten Artikel ist in Tab. 1 dargestellt.

**Tab. 1:** Publikationen mit inhaltlich beschriebener edukativer Intervention bei Medikamentenübergebrauch oder chronischen Kopfschmerzen durch Übergebrauch von Schmerz- und Migränemitteln

Referenz	Art der Publikation	Zentrale Ergebnisse
Carlsen et al. 2018 [2]	Awareness-Kampagne	Wissen über MOH ↑ (von 31 % auf 38 %)
Fritsche et al. 2010 [5]	RCT: Minimalkontaktprogramm basierend auf kognitiver Verhaltenstherapie vs. Broschüre	Beide Maßnahmen wirksam, MHD, MMD und MID ↓ gegenüber Baseline (signifikant), kein Patient entwickelte MOH
Lagman-Bartolome et al. 2018 [14]	Prospektive Prä-post-Interventionsstudie: HEAD-Überbrückungsprogramm während Wartezeit auf einen Termin	Medikamentenübergebrauch ↓ (signifikant), Lifestyle-Faktoren ↑
Kristoffersen et al. 2012 [9]	RCT: „brief intervention (BI)“ vs. „business as usual (BAU)“	MHD und MID ↓ (signifikant) in BI-Gruppe im Vergleich zu BAU [11]
Lake 2006 [15]	Narrativer Review	Vorschläge für Edukationsinhalte
McLean et al. 2020 [17]	Evaluierung eines Programms zur Patientenedukation	Weiterempfehlung bei 87 % der Befragten; hilfreichste Themen u. a. Medikation, Managementansätze und Trigger
Moraes Alves et al. 2021 [18]	RCT: FRAMES-Programm vs. unstrukturierte Edukation	Beide Maßnahmen vergleichbar wirksam, MHD und MID ↓ (signifikant) gegenüber Baseline
Mose et al. 2021 [19]	RCT: Patientenedukationsprogramm (Motivations- und Bewältigungsstrategien) vs. Standardbehandlung	Beide Maßnahmen vergleichbar wirksam, MHD↓ (signifikant) gegenüber Baseline [20]
Pijpers et al. 2022 [22]	RCT: maximale vs. minimale Verhaltensintervention während der Medikamentenpause	Beide Maßnahmen: MMD ↓ (signifikant); Patienten mit maximaler Intervention nahmen signifikant seltener Akutmedikamente ein als mit minimaler Intervention
Rossi et al. 2006 [23]	Offene RCT: Beratung vs. strukturiertes Entgiftungsprogramm	Edukation war vergleichbar wirksam wie Medikamentenentzug, Behandlungserfolg bei 75,4 % der gesamten Kohorte
Steiner et al. 2019 [24]	Konsensusartikel der EHF und der globalen Kopfschmerzkampagne „Lifting The Burden“	Entwicklung von Edukationsmaterial zu MOH
TOP Headache Working Group 2016 [26]	Klinische Praxisleitlinie zur Behandlung von Kopfschmerzen in der Primärversorgung (2016, 2. Auflage)	Informationen für Edukation bei MOH

*EHF* European Headache Federation, *FRAMES-Technik* „**f**eedback about personal risk, **r**esponsibility of patient, **a**dvice to change, **m**enu of options, **e**mpathy, **s**elf-efficacy enhancement“, *GP* „general practitioner“, *HEAD Program* Headache Education Active-Waiting Directive Program, *MHD* „monthly headache days“, *MID* „medication intake days“, *MMD* „monthly migraine days“, *MOH* chronische Kopfschmerzen durch Übergebrauch von Schmerz- und Migränemitteln, *RCT* randomisierte, kontrollierte Studie, *TOP* „toward optimized practice“

Sieben der zwölf Artikel waren kontrollierte Studien und wiesen Edukation als wirksames Instrument zur Reduktion oder Prävention von Medikamentenübergebrauch oder chronischen Kopfschmerzen durch Übergebrauch von Schmerz- und Migränemitteln nach [5, 11, 14, 18, 20, 22, 23]. Hierbei wurden in den verschiedenen Studien unterschiedliche Ansätze untersucht (Tab. 1). Unstrukturierte Beratungen waren in vergleichenden Studien ähnlich wirksam wie strukturierte Beratungen [5, 18, 20]. Bei den fünf übrigen Publikationen handelte sich um eine allgemeine Leitlinie zu Kopfschmerztherapie, Übersichtsartikel oder unkontrollierte Studien (Tab. 1; [2, 15, 17, 24, 26]).

Alle beschriebenen Edukationsansätze wurden auf ihre inhaltlichen Schwerpunkte hin analysiert. Wir identifizierten vier übergeordnete Themenbereiche (Tab. 2), die nachfolgend näher dargestellt werden.

**Tab. 2:** Qualitative inhaltliche Analyse der edukativen Interventionen

Themenbereich	Inhaltliche Schwerpunkte	Zahl der Nennungen	Referenzen
Grundlagen zum Konzept Medikamentenübergebrauch oder chronischen Kopfschmerzen durch Übergebrauch von Schmerz- und Migränemitteln	Zusammenhang zwischen akuter Medikamenteneinnahme, Kopfschmerzen und Chronifizierung	*12*	[2, 5, 9, 14, 15, 17–19, 22–24, 26]
	Medikamentenpause kann zu Kopfschmerzen und anderen Entzugserscheinungen führen	*12*	[2, 5, 9, 14, 15, 17–19, 22–24, 26]
	Obergrenze der Einnahme von Akutmedikation	*12*	[2, 5, 9, 14, 15, 17–19, 22–24, 26]
	Kopfschmerzkalender und/oder PRO	*11*	[2, 5, 9, 14, 15, 17, 18, 22–24, 26]
	Vorteile einer reduzierten Medikamenteneinnahme	*4*	[9, 18, 23, 26]
Risikofaktoren	Aufklärung über generelle Risikofaktoren, Identifizierung und Berücksichtigung persönlicher Risikofaktoren	*8*	[5, 9, 14, 15, 18, 22, 24, 26]
Therapieoptionen	Modifikation von Verhaltensfaktoren für einen besseren Umgang mit Attacken (u. a. Migräneaktionsplan, Bewältigungsstrategien, z. B. bei Attackenangst)	*8*	[5, 14, 15, 17, 19, 22, 24, 26]
	Lifestyle-Modifikationen (interne + externe Faktoren, u. a. Stressmanagement, Trigger, Ernährung, Sport, Schlaf)	*8*	[5, 14, 17–19, 22, 24, 26]
	Akutmedikation (u. a. Therapieoptionen/-optimierung/-pläne)	*7*	[5, 9, 17, 18, 23, 24, 26]
	Medikamentöse Prophylaxe (Therapieoptionen/-pläne/-optimierung)	7*	[2, 5, 9, 15, 17, 24, 26]
	Nichtmedikamentöse Therapieoptionen (PMR, KVT, Biofeedback)	*4*	[5, 17, 22, 24]
Absprache von Behandlungszielen	Patienten-Follow-up	*9*	[5, 9, 14, 18, 19, 22–24, 26]
	Eigenverantwortliches Management (u. a. Selbstreflexion, Selbstwirksamkeit)	*6*	[9, 14, 18, 19, 23, 26]
	Kooperatives Behandlungsmodell (u. a. einfühlsame Kommunikation, gegenseitiges Verständnis)	*4*	[9, 15, 18, 19]
	Motivationstechniken	*3*	[18, 19, 22]
	Wertebasierte Ansätze	*2*	[19, 22]
	Erwartungsmanagement	*2*	[14, 15]

*KVT* kognitive Verhaltenstherapie, *MOH* chronische Kopfschmerzen durch Übergebrauch von Schmerz- und Migränemitteln, *PMR* progressive Muskelrelaxation, *PRO* „patient-reported outcomes“

*Aus Pijpers et al. 2022 [22] und Rossi et al. 2006 [23] geht nicht eindeutig hervor, ob über medikamentöse Prophylaxe im Rahmen der Edukation im Allgemeinen aufgeklärt wurde; beide Studien wurden an dieser Stelle deshalb nicht gezählt

### Grundlagen zum Konzept Medikamentenübergebrauch oder chronischen Kopfschmerzen durch Übergebrauch von Schmerz- und Migränemitteln

Die Aspekte „Zusammenhang zwischen akuter Medikamenteneinnahme, Kopfschmerzen und Chronifizierung“, „Medikamentenpause“ und „Obergrenze der Einnahme von Akutmedikation“ waren Inhalt aller beschriebenen Interventionen. Kopfschmerzkalender wurden in 11 Interventionen besprochen [2, 5, 9, 14, 15, 17, 18, 22–24, 26]. Dahingegen wurden Vorteile einer reduzierten Medikamenteneinnahme in nur 4/12 Artikeln thematisiert [9, 18, 23, 26]. Genannte Vorteile waren Reduktion der Schmerzempfindlichkeit, bessere Wirksamkeit der Medikation, weniger Nebenwirkungen, geringere Medikationskosten, bessere Lebensqualität und bessere Diagnostizierbarkeit der primären Kopfschmerzerkrankung [9, 23].

### Risikofaktoren

Risikofaktoren für chronische Kopfschmerzen durch Übergebrauch von Schmerz- und Migränemitteln wurden im Rahmen von 8 Interventionen besprochen [5, 9, 14, 15, 18, 22, 24, 26]. Hierbei lassen sich generelle Risikofaktoren und patientenindividuelle Risikofaktoren unterscheiden.

### Therapieoptionen

In 8 Publikationen wurden Therapieoptionen im Rahmen der Edukation besprochen. Diese umfassten u. a. die Modifikation von Verhaltensfaktoren im Umgang mit Attacken, Lifestyle-Modifikationen, Optimierung der Akutmedikation, Medikation zur Prophylaxe sowie nichtmedikamentöse Therapieoptionen. Drei von acht Publikationen deckten das gesamte Spektrum an Therapieoptionen ab [5, 17, 24], vier Publikationen nannten nichtmedikamentöse Optionen [5, 17, 22, 24].

### Absprache von Behandlungszielen

In 9 Publikationen wurde beschrieben, dass mit den Patienten Follow-up-Termine zur Nachsorge vereinbart wurden [5, 9, 14, 18, 19, 22–24, 26]. Eigenverantwortliches Management der Patienten unter Einbeziehung von Selbstreflexion und Selbstwirksamkeit wurde in 6 Studien thematisiert [9, 14, 18, 19, 23, 26]. Andere Themenbereiche wie z. B. kooperative Behandlungsansätze [9, 15, 18, 19] und Motivationstechniken [18, 19, 22] kamen seltener vor.

## Diskussion

Patienten mit chronischen Kopfschmerzen durch Übergebrauch von Schmerz- und Migränemitteln leiden unter starken Einschränkungen und reduzierter Lebensqualität [1]. Außerdem ist der Übergebrauch von Akutmedikation ein Risikofaktor für eine Krankheitsprogression [16]. Darum ist es wichtig, Patienten dabei zu unterstützen, die Einnahme von Akutmedikation zu reduzieren. Der empfohlene Therapiealgorithmus in der aktuellen Leitlinie zu Kopfschmerz bei Übergebrauch von Schmerz- oder Migränemitteln sieht hierfür als Maßnahme u. a. eine Patientenedukation vor [4].

Die Auswertung der hier analysierten publizierten Edukationsansätze zeigte, dass Patientenedukation uneinheitlich umgesetzt wird. Bestimmte Inhalte waren häufig, andere nur selten Teil einer Edukation (Tab. 2). So wird die Darstellung des Zusammenhangs zwischen akuter Medikamenteneinnahme, Kopfschmerzen und Chronifizierung oft aufgeführt, ebenso wie die Effekte einer Medikamentenpause und die Beschreibung der Obergrenze der Menge an Akutmedikation. Weniger häufig dagegen werden die Vorteile der reduzierten Medikamenteneinnahme hervorgehoben. Trotzdem mag es sinnvoll sein, einige der selten angesprochenen Themengebiete bzw. Methoden in einen Standardleitfaden aufzunehmen. Die zahlenmäßig meisten Themenbereiche werden im FRAMES-Programm [18], der Leitlinie der Toward Optimized Practice Working Group [26] und der Brief-intervention-Methode von Kristoffersen et al. [9] adressiert (jeweils 11/12).

Im Folgenden werden die Inhalte der beschriebenen Edukationsansätze nach Themenbereichen gruppiert diskutiert. Da dies insgesamt sehr viele Themen umfasst, wird jedes einzelne nur kurz besprochen.

### Grundlagen zum Konzept Medikamentenübergebrauch oder chronischen Kopfschmerzen durch Übergebrauch von Schmerz- und Migränemitteln

#### Zusammenhang zwischen akuter Medikamenteneinnahme, Kopfschmerzen und Chronifizierung

Dass ein Zusammenhang zwischen der übermäßigen Medikamenteneinnahme und der Verstärkung von Kopfschmerzen bestehen kann, ist vielen Patienten nicht bekannt [1] und sollte deshalb geschult werden. Es ist wichtig, bei der Therapie den Patienten mit Medikamentenübergebrauch oder chronischen Kopfschmerzen durch Übergebrauch von Schmerz- und Migränemitteln zu vermitteln, dass sie nicht selbst schuld an dem Übergebrauch von Akutmedikation sind. Eine Stigmatisierung sollte vermieden werden [27].

#### Medikamentenpause kann verstärkt zu Kopfschmerzen und anderen Entzugserscheinungen führen

Bei chronischen Kopfschmerzen durch Übergebrauch von Schmerz- und Migränemitteln kann eine Medikamentenpause sinnvoll sein. Diese kann initial verstärkt zu Kopfschmerzen und anderen Entzugserscheinungen führen, worüber der Patient aufgeklärt werden sollte [9].

#### Obergrenze der Einnahme von Akutmedikation

Es ist wichtig, auf die empfohlene Obergrenze für die Einnahme von Akutmedikation hinzuweisen [4], die vielen Patienten möglicherweise nicht bekannt ist.

#### Kopfschmerzkalender und/oder PRO

Um einen Medikamentenübergebrauch oder chronische Kopfschmerzen durch Übergebrauch von Schmerz- und Migränemitteln festzustellen, ist es wichtig, einen Kopfschmerzkalender zu führen. Zusätzlich können klinische Skalen zu patientenberichteten Outcomes (PRO) in das Gespräch einfließen. In den gefundenen Ansätzen wurden der Headache Impact Test‑6 [18] und die Severity of Dependence Scale [9] verwendet. Um die Lebensqualität der Patienten zu erfassen, wird auch der Migraine-Disability-Assessment-Fragebogen (MIDAS) in der Leitlinie empfohlen [4].

### Risikofaktoren

Modifizierbare Risikofaktoren für chronische Kopfschmerzen durch Übergebrauch von Schmerz- und Migränemitteln sind körperliche Inaktivität, Komorbiditäten wie Depression oder Angsterkrankungen, andere chronische Schmerzerkrankungen, Übergewicht, Rauchen, Suchtgefährdung und Stress [4]. Eine Diskussion mit dem Patienten über individuelle Risikofaktoren kann Wege aufzeigen, diese zu modifizieren oder zu eliminieren. Auch Angst vor Attacken kann ein Risikofaktor für chronische Kopfschmerzen durch Übergebrauch von Schmerz- und Migränemitteln sein, da Patienten gegebenenfalls bereits im Vorfeld möglicher Attacken Medikation einnehmen [8].

### Therapieoptionen

#### Verhaltensmodifikationen und Lifestyle-Modifikationen

Themen aus dem Gebiet der Lifestyle- oder Verhaltensmodifikationen waren in der analysierten Literatur beispielsweise das Management von Auslösern, Ernährungs- und Trinkverhalten, Schlafhygiene, körperliche Aktivität, Entspannungsverfahren und Stressbewältigung [17]. Hinweise auf externe (z. B. Lagerhaltung von Medikamenten) und interne (z. B. Angst vor Attacken, Stressniveau) Einflussfaktoren auf die Medikamenteneinnahme können das Bewusstsein schärfen und zu Verhaltensänderungen verhelfen. Auch Bewältigungsstrategien bei antizipatorischer Angst vor Migräneattacken wurden thematisiert [5]. Diagnostische Verfahren sowie Behandlungsoptionen bei Attackenangst werden von Klan et al. [8] thematisiert. Bewältigungsstrategien und Motivation zur Verhaltensänderung haben Patienten, die an einer Intervention im Rahmen einer RCT teilnahmen, als besonders relevant erlebt [19].

#### Akutmedikation

Optimierung der Akutmedikation kann helfen, den Gebrauch zu verringern. Dies kann z. B. die Erstellung eines Therapieplans mit genauer Anleitung zur Anwendung umfassen.

#### Medikamentöse Prophylaxe

Patienten mit Risiko für Medikamentenübergebrauch oder chronischen Kopfschmerzen durch Übergebrauch von Schmerz- und Migränemitteln wird eine medikamentöse Prophylaxe empfohlen. Wirksamkeitsnachweise bei der Prophylaxe bei Migräne mit begleitenden chronischen Kopfschmerzen durch Übergebrauch von Schmerz- und Migränemitteln wurden für Topiramat, Onabotulinumtoxin A und die CGRP- und CGRP-Rezeptor-Antikörper erbracht [4, 25]. Die Kombination aus Edukation und medikamentöser Prophylaxe wird aktuell in der RESOLUTION-Studie untersucht [7]. Patienten mit CM und chronischen Kopfschmerzen durch Übergebrauch von Schmerz- und Migränemitteln werden dort auf eine evidenzgestützte [10, 11] Edukation nach Kristoffersen et al. („brief intervention“; [9]) in Kombination mit dem Anti-CGRP-Antikörper Eptinezumab oder Placebo randomisiert [7].

#### Nichtmedikamentöse Therapieoptionen

Verhaltenstherapeutische Ansätze können eigenständig oder in Kombination mit medikamentöser Behandlung wirksam sein [13]. Sie können die Kopfschmerzhäufigkeit reduzieren, psychische Beeinträchtigungen lindern und zu besserer Akzeptanz und Attackenkontrolle führen [21].

### Absprache von Behandlungszielen

Beim Themenfeld „Absprache von Behandlungszielen“ sind Gesprächsstrategien im Fokus. Solche Strategien können die Edukationsinhalte patientenorientiert erweitern.

#### Patienten-Follow-up

Es ist wichtig, engmaschig Verlaufskontrollen durchzuführen. Bei diesen Follow-up-Terminen kann der Erfolg evaluiert und das weitere Vorgehen besprochen werden.

#### Eigenverantwortliches Management

Durch eigenverantwortliches Management kann die Selbstwirksamkeit der Patienten gefördert werden.

#### Kooperatives Behandlungsmodell und Motivationstechniken

Eine partizipative Entscheidungsfindung zwischen Behandelndem und Patient wurde nur selten thematisiert. Sie könnte für die Adhärenz der Patienten eine wichtige Rolle spielen. „Motivational interviewing“ könnte als strukturierte Motivationstechnik ein geeigneter Ansatz sein [19]. Gegenseitiges Verständnis und eine partizipative Entscheidungsfindung können die Motivation zur Verhaltensänderung fördern [9].

Die Berücksichtigung von Patientenfeedback [17, 19] kann dabei helfen, die Relevanz bestimmter Maßnahmen besser zu verstehen und die Partizipation und Adhärenz der Patienten zu steigern.

#### Wertebasierte Ansätze

Wird mit dem Patienten ergründet, wie die eigenen Werte und Gefühle Verhaltensentscheidungen beeinflussen, kann dies das eigenverantwortliche Management fördern [19].

#### Erwartungsmanagement

Es ist wichtig, mit dem Patienten über Zeitrahmen und Ausmaß der zu erwartenden Verbesserungen zu sprechen, um keine überhöhten Erwartungen zu wecken [12]. Erwartungseffekte können sich negativ auf das Behandlungsergebnis auswirken [12].

In der Zusammenschau bieten sich sehr unterschiedliche Ansätze, mit denen chronische Kopfschmerzen durch Übergebrauch von Schmerz- und Migränemitteln behandelt werden können. Dabei kann davon ausgegangen werden, dass einfacher umzusetzende Therapien zu schnelleren und damit auch zu effizienteren Ergebnissen führen. So sollte eine Darstellung der Grundlagen zum Konzept von chronischen Kopfschmerzen durch Übergebrauch von Schmerz- und Migränemitteln in Form eines schriftlichen Ratgebers überreicht werden. Dieser sollte auch die Anleitung zum Führen eines Kopfschmerzkalenders beinhalten. Auch die Absprache von Behandlungszielen kann im Patientengespräch umgesetzt werden.

Problematischer ist die Kontrolle von modifizierbaren Risikofaktoren bei der Behandlung von chronischen Kopfschmerzen durch Übergebrauch von Schmerz- und Migränemitteln. Die Modifikation von Lebensstilfaktoren wie Rauchen und Übergewicht ist schwierig, da es sich zum Teil um Abhängigkeiten handelt. Bei manifesten psychischen Erkrankungen wie beispielsweise einer Depression oder einer Angststörung sollte eine interdisziplinäre Vorgehensweise erfolgen.

Als Grundlage für eine Broschüre bieten sich die Darstellung der Mechanismen von chronischen Kopfschmerzen durch Übergebrauch von Schmerz- und Migränemitteln und den Zusammenhang mit Lebensstilfaktoren als Risikofaktoren und die Selbstbeobachtung durch ein Kopfschmerztagebuch an.

## Limitationen

Zur Auswertung wurden die Edukationsinhalte qualitativ kategorisiert. Trotz methodischen Vorgehens kann dies einem subjektiven Bias unterliegen.

## Ausblick

Auf der Grundlage der hier vorliegenden Information können Empfehlungen für einen Leitfaden erarbeitet werden, welcher Behandelnden hilft, strukturierte Edukationen ihrer Patienten mit chronischen Kopfschmerzen durch Übergebrauch von Schmerz- und Migränemitteln durchzuführen.

## Fazit für die Praxis

- Patienten mit chronischen Kopfschmerzen durch Übergebrauch von Schmerz- und Migränemitteln benötigen Unterstützung, um ihren Übergebrauch zu beenden.
- Eine Edukation des Patienten kann dafür bereits ausreichend sein.
- Verschiedene Ansätze sind publiziert und können als Grundlage dienen.
